# Loss of *DMRT1* gene in a Mos 45,XY,-9[8]/46,XY,r(9)[29]/47,XY,+idic r(9)× 2[1]/46,XY,idic r(9)[1]/46,XY[1] female presenting with short stature

**DOI:** 10.1186/s13039-018-0379-z

**Published:** 2018-05-08

**Authors:** Bagas A. Marsudi, Hannie Kartapradja, Chrysantine Paramayuda, Jose R. L. Batubara, Alida R. Harahap, Nanis S. Marzuki

**Affiliations:** 1 Eijkman Institute for Molecular Biology, Jakarta, Indonesia; 2 Department of Child Health, Faculty of Medicine, Universitas Indonesia, Jakarta, Indonesia

**Keywords:** *DMRT1*, Sex-reversal, 46,XY-DSD, Ring-chromosome-9, Short-stature

## Abstract

**Background:**

A 46,XY sex reversal syndrome is characterized by discordant genetic and phenotypic sex, leading to normal external female genitalia, undeveloped gonads and presence of Müllerian structures in an otherwise 46,XY individual. Chromosome 9pter aberrations, such as ring chromosome have been reported to cause 46,XY disorders of sex development (DSD), due to involvement of *DMRT1* gene located at the 9p24.3 region.

**Case presentation:**

This study presents a unique case of a 12-year-old female with mos 46,XY, (r)9[31]/45,XY,-9[9] karyotype, presenting with intellectual disability and short stature, mimicking Turner syndrome. Re-karyotyping was performed using standard GTL-banding technique. Further cytogenetic study using standard metaphase fluorescent in situ hybridization (FISH) technique was applied to cultured lymphocytes from peripheral blood, hybridized using green control probe specific to 9q21 loci, and red *DMRT1* probe specific to 9p24.3 loci. Cytogenetics and FISH analysis revealed mos 45,XY,-9[8]/46,XY,r(9)[29]/47,XY,+idic r(9)× 2[1]/46,XY,idic r(9)[1]/46,XY[1] and haploinsufficiency of *DMRT1* gene in most cells. CGH array revealed a deletion around 1.25 Mb at 9p24.3 loci [arr 9p24.3(204,193-1,457,665)× 1] and three duplications around 13 Mb [9p24.3p22.3(1,477,660-14,506,754)× 3] near the breakage point that formed the ring chromosome 9.

**Conclusions:**

The clinical presentation of the subject that mimics Turner syndrome highlights the importance of cytogenetic analysis to detect the possibility of ring chromosome 9. Sex reversal due to haploinsufficiency of *DMRT1* gene in ring chromosome 9 structures is exceedingly rare with only a handful of cases ever reported. This finding further highlights the importance of *DMRT1* gene in sex determination and differentiation in males. More research is required to pinpoint the exact mechanism that underlies sex reversal caused by *DMRT1* haploinsufficiency.

## Background

Sex determination and development in males occurs during the embryonic period, and is initiated by the expression of *SRY* gene located on the short arm of the Y chromosome. Expression of *SRY* gene upregulates, and acts in synergy with, several other genes such as *SOX9*, *NR5A1*, *FGF9*, *GATA4*, *WT1*, *DMRT1*, and *ATRX*. Defects affecting the aforementioned genes may lead to sex reversal or underdeveloped male sex phenotypes [1, 2].

XY sex reversal is a relatively rare type of DSD (disorder of sexual development). Elaborating its molecular defects play an imperative role in understanding sex determination and development in the embryo. The incidence of XY DSD is found to be 1 in 20,000 live births and is diagnosed with the presence of a female phenotype or undervirilized male in XY individuals [2]. Despite genetic studies, only 15-30% of XY DSD cases have specific identified etiologies [3].

Chromosome 9 aberrations involving the terminal end of p arm, such as terminal p deletions or ring chromosomes have been reported to cause 46,XY DSD. This terminal region of chromosome 9p (9p24.3 region) contains 3 types of *DMRT* genes (*DMRT1*-*3*). *DMRT* (Double sex and mab-3 related transcription factor) has a DNA binding domain with fingerlike zinc ions [4]. Among those types of DMRT genes, *DMRT1* is exclusively and highly expressed in the genital ridge and Sertoli cells [5]. Therefore, *DMRT1* is suggested to have an important role in sex differentiation.

To our knowledge, there have been very few cases of XY DSD caused by *DMRT1* gene abnormality reported. In addition, the pathomechanism has not been fully understood. Therefore, we conducted a case study of a 46,XY female to better understand the genotypic and phenotypic characteristics related to 9p24.3 aberration and *DMRT1* loss.

## Case presentation

The patient is the first child of non-consanguineous parents with an ordinary family history, as well as uncomplicated pregnancy and delivery. The patient was born at term with birth weight of 2850 g and length of 47 cm. Normal female external genitalia was observed and the child was identified as female. At the age of 20 months she was diagnosed with tuberculosis and malnourished which were promptly treated. Since she was 6 months-old she had growth faltering, and at 18-months-old, height for age was consecutively below 3rd percentile (NCHS-CDC 2000). There was no catch up growth despite tuberculosis treatment and appropriate diet. She was hypotonic and her developmental milestones were also delayed. She started crawling at 9 months, walking at 16 months and babbling which persisted until 18 months of age in addition to poor progression of speech ability. She was stated to have Sensory Integration Disorder (SID) and underwent speech therapy. At the age of 2-years-old her bone age was delayed (9 months).

The patient was completely evaluated at 5 years and 2 months of age. Clinical examination disclosed a height of 99 cm (below 3rd percentile), weight of 17.5 kg (below 85th percentile) and BMI 17.85 kg/m^2^ (below 50th percentile). She was referred for karyotyping due to short stature. Karyotyping results showed mos 46,XY,r(9)[31]/45,XY,-9[9]. Patient did not undergo any therapy due to parental refusal.

At the age of 12 years and 8 months old she was reassessed for more detailed evaluation on *DMRT1* gene. Based on parental observation, the patient had some degree intellectual disability and poor school performance. Patient also had difficulty in socializing with classmates of the same age, choosing instead to play with toddlers and children of younger age. Currently, the patient is being home schooled. Her body weighed 46.5 kg (between 75th and 50th percentile), a measured height of 133.6 cm (below 3rd percentile) and BMI of 26.06 kg/m^2^, indicating obesity. General physical examination showed short stature, whorled pattern hypopigmentation on lower arms, hypertelorism, epicanthal fold, down slanted palpebral fissures, high palate, long philtrum, short neck, cubitus valgus, clinodactyly of 5th finger and broad chest. No intrabdominal or inguinal mass was detected. Genital examination revealed normal female external genitalia, with pubertal state of A1M1P1 (Tanner staging).

Standard karyotyping analysis was performed using metaphase chromosome preparations from phytohaemagglutinin-stimulated blood lymphocytes. The metaphases were stained using conventional GTL-banding technique. Chromosome analysis was done under light microscope with 100× magnification. A total of 40 metaphases were analyzed. The analysis and nomenclature of the chromosome were based on ISCN 2013 [6].

**Fig. 1:**
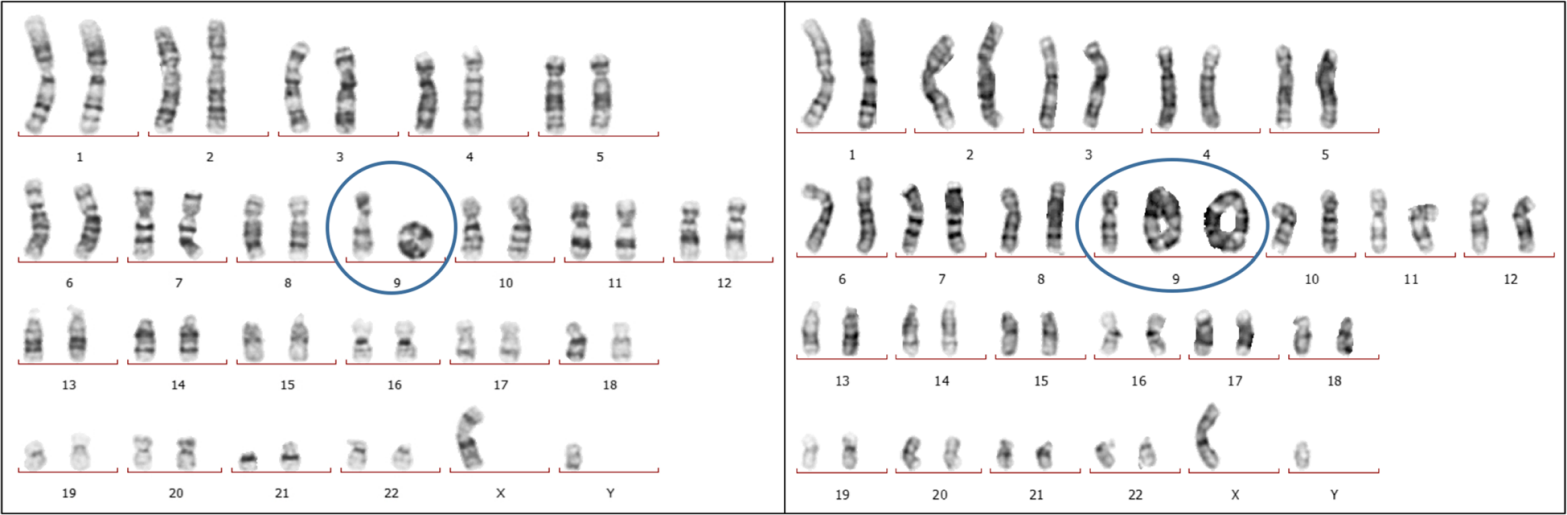
Karyotype results of 46,XY,r(9) (shown left) and 46,XY,idic r(9)× 2 (right)

**Fig. 2:**
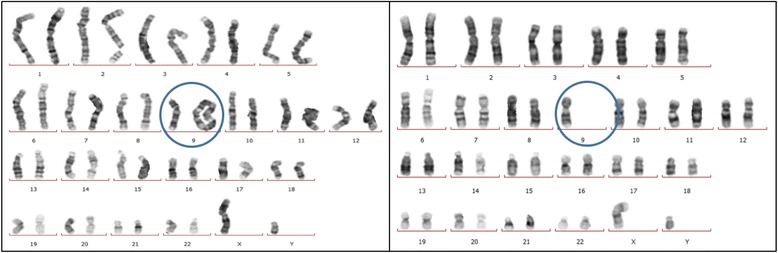
Karyotype results containing isodicentric ring chromosome 9 (left) and the other with monosomy 9 (right)

Re-karyotyping revealed new findings (Figs. 1 and 2). A total of 40 metaphase preparations were analysed at the 550-band level according to ISCN 2013 [6]. New cell lines were detected revealing karyotype of mos 45,XY,-9[8]/46,XY,r(9)[29]/47,XY,+idic r(9)× 2[1]/46,XY,idic r(9)[1]/46,XY[1].

**Fig. 3:**
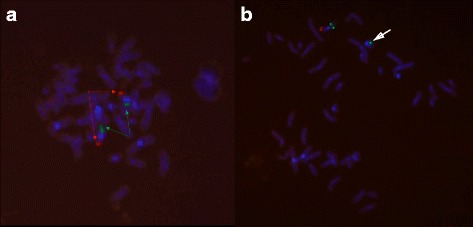
Panel **a** Normal 46 XY with both control (green) and *DMRT1*(red) probe signals detected. Panel **b** Ring chromosome 9 without DMRT 1 signal

**Fig. 4:**
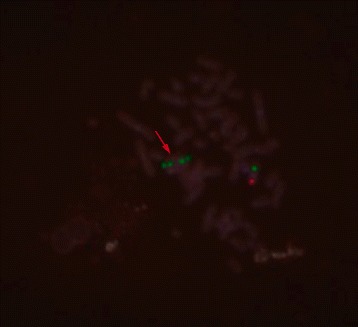
Isodicentric ring chromosome 9 with absence of *DMRT1* signal (arrow)

**Fig. 5:**
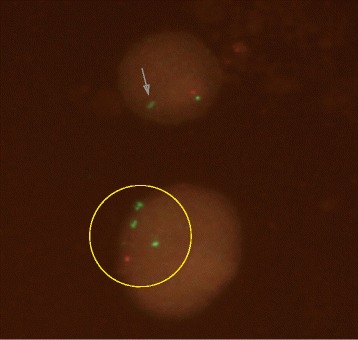
Interphase preparation showing two cells without one copy of *DMRT1* signal (arrow) and isodicentric ring shown by detection of two adjacent green control probes (cirlce)

Metaphase FISH for *DMRT1* was conducted using standard procedure according to probe manufacturer (Empire Genomics, USA). Green control probe hybridized to 9q21 loci, and red *DMRT1* probe hybridized to 9p24.3 loci. Analysis was performed on 65 metaphases under fluorescence microscopy with 100× magnification. Images were captured using NIS Elements Basic Research Imaging Software (Nikon Corporation). Quality and signal strength for all probes across the majority of cells were adequate after optimization. Metaphase FISH analysis on 65 metaphase chromosomes revealed mosaicism. Fifty six cells showed ring chromosome 9, 15 monosomy 9, 2 isodicentric ring 9, and 2 with normal chromosome 9. Except the two normal chromosome 9 (Fig. 3a), all other cells showed only one *DMRT1* signal (Figs. 3b, 4 and 5). This confirmed haploinsufficiency of *DMRT1* gene in most cells. Thus the patient karyotype was mos 45,XY.ish del(9)(q24.3)(DMRT1+,9q21+)[56]/46,XY.ish r(9)(q24.3) (DMRT1+,9q21++)[15]/47,XY,+r(9)× 2.ish, r(9)(q24.3)(DMRT1+,9q21+++)[2]/46,XY.ish(9)(q24.3)(DMRT1++, 9q21++)[2].

**Fig. 6:**
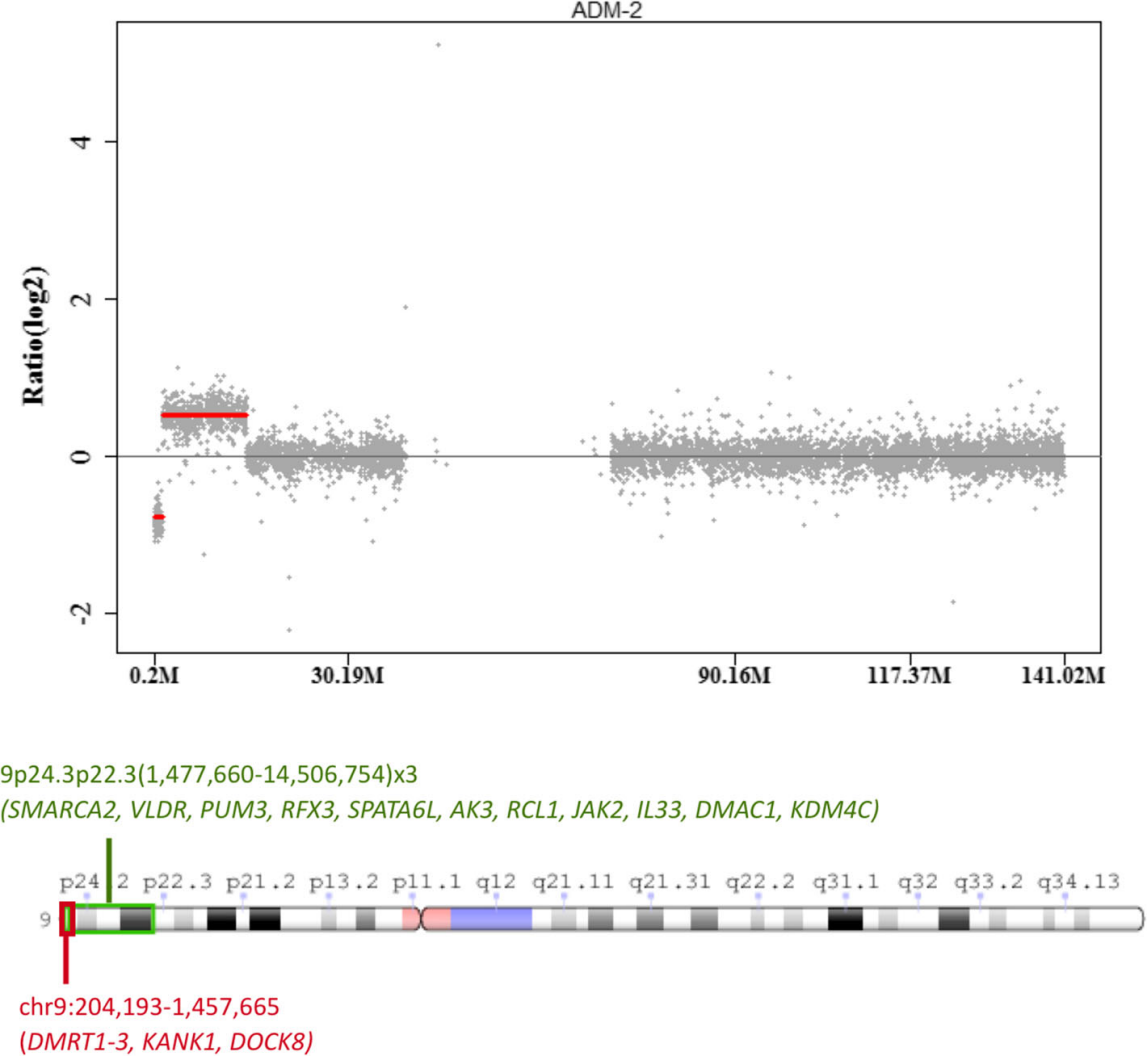
Array-CGH log2 ratio profile of our case showing and an ideogram of chromosome 9 showing the deleted region (red) and duplicated region (green), the mutated genes are also noted

For CGH array, DNA labeling and hybridization were performed by using the Agilent Oligonucleotide Array-Based CGH for Genomic DNA Analysis protocol (V 7.3, 2014). Labeled Test (Cy5) and Reference (Cy3) DNA samples were paired and co-hybridized to the SurePrint G3 Human CGH Microarrays, 4 × 180 K (Agilent®) at 67 °C, 20 rpm for 24 h, then washed at room temperature by using the Agilent Oligonucleotide Array-Based CGH for Genomic DNA Analysis protocol (V7.3, 2014). The hybridized array was immediately scanned with an Agilent Microarray Scanner (Agilent Technologies, Inc.). CGH array revealed a deletion around 1.25 Mb at 9p24.3 loci [arr 9p24.3(204,193-1,457,665)× 1] and three duplications around 13 Mb at the subtelomeric region near the breakage point that formed the ring chromosome 9 [9p24.3p22.3(1,477,660-14,506,754)× 3]. The deletion encompassed *DMRT1-3* genes, in addition to *KANK1* and *DOCK8* genes, while the 13 Mb duplication encompassed *SMARCA2, VLDR, PUM3, RFX3, SPATA6L, AK3, RCL1, JAK2, IL33, DMAC1, KDM4C* genes (Fig. 6).

## Discussion

To the best of our knowledge there have been a total 98 reported cases of terminal 9p aberrations, but only 9 have tried to detect the presence of *DMRT1* and correlation with XY DSD and only very few cases resulted in sex reversal [7–11]. Confirmation of *DMRT1* gene loss in other reports were conducted using several methods including, CGH microarray, metaphase FISH using 9p terminal probes, and MLPA [8, 11]. In our study we opted metaphase FISH using probe specific to *DMRT1* gene in order to explain the sex reversal presented in the patient, as this technique is less laborious and affordable.

In our case, patient was refered for karyotyping due to short stature and other signs suggesting of Turner syndrome. A meta-analysis found that Turner syndrome (TS) commonly presents with short stature, puberty development abnormality, edema of hands or feet, nuchal folds, left-sided cardiac anomalies, low hairline, low set ears, small mandible, cubitus valgus, and speech delay [12]. In our case, Turner syndrome was in accordance with most of the symptoms, although global development delay is uncommon in girls with TS [13]. With the finding of XY karyotype without X mosaicism, TS was excluded, but it is interesting to note the overlapping phenotypes of TS and ring chromosome 9 syndrome. The phenotype similarities further highlights the importance of early karyotyping in such cases to establish the specific etiology.

Our case also presented with motoric and speech development delay, along with psychosocial problems. Presence of ring chromosome abnormality is frequently associated with a “ring syndrome”, characterized by growth and development abnormalities [14, 15]. A study by Kosztolányi et al. [15], revealed ring syndrome occurred in 40 out of the 207 ring chromosome cases. It is theorized that ring syndrome is due to chromosome instability during cell division subsequently causes cell death in many organs, causing growth and developmental delays [14]. This is further proven with the occurrence of ring syndrome in complete ring structures (fusion of telomeres without loss of genetic material). Another mechanism involves telomere-to-telomere fusion that produces a pseudo-complete ring with cryptic deletions of subtelomeric genes [16].

The clinical appearance reported in our patient can also be due to loss of sub-telomeric gene loss. A study by Mundhofir, FEP et al. [17], found mild to moderate intellectual disability, developmental delay and short stature in all cases of subtelomeric rearrangements. The same study also had varied findings of hypertelorism, hypotonia, and clinodactyly of 5th finger, which was present in our patient. In addition, studies of distal 9p segment deletion has been linked to mental retardation, motoric development abnormality, speech delay/language abnormality, trigonocephaly and sex reversal [8], which also matches with the patient history and symptoms. The combination of ring instability, subtelomeric genes loss and terminal 9p deletion explains the clinical findings in our patient. Besides this, partial 9p trisomy as found in CGH array analysis could be responsible for the phenotype found in our case.

The occurrence of mosaicism in this patient can be of two mechanisms. The first possible mechanism is the generation of an abnormal cell line during early mitotic cycle [18]. Secondly, the conceptus starts with supernumerary chromosomes, which are subsequently lost during mitosis [18]. The presence of a normal cell line in our patient infers that the mutation occurred in early mitosis that generated the ring chromosome, which then produced many other abnormal cell lines. The presence of a normal cell line can also be due to somatic reduplication. However, this does not explain the presence of isodicentric chromosome 9 cell line. The presence of the isodicentric ring chromosome 9 population is better explained by the natural process of ring chromosome cell division.

Ring chromosomes are a rare chromosomal abnormality with a frequency of 1:60.000 cases [19] and have been found in all autosomal and sex chromosomes where 50% are derived from acrocentric chromosomes [20]. Different phenotypes manifest according to the lost genetic material and degree of ring instability [21]. To our knowledge, this is the first reported ring chromosome 9 case from Indonesia. In other reported cases, breakage point was usually found at p22-24 and q33-34, but end to end telomeric fusion has also been reported [22].

**Fig. 7:**
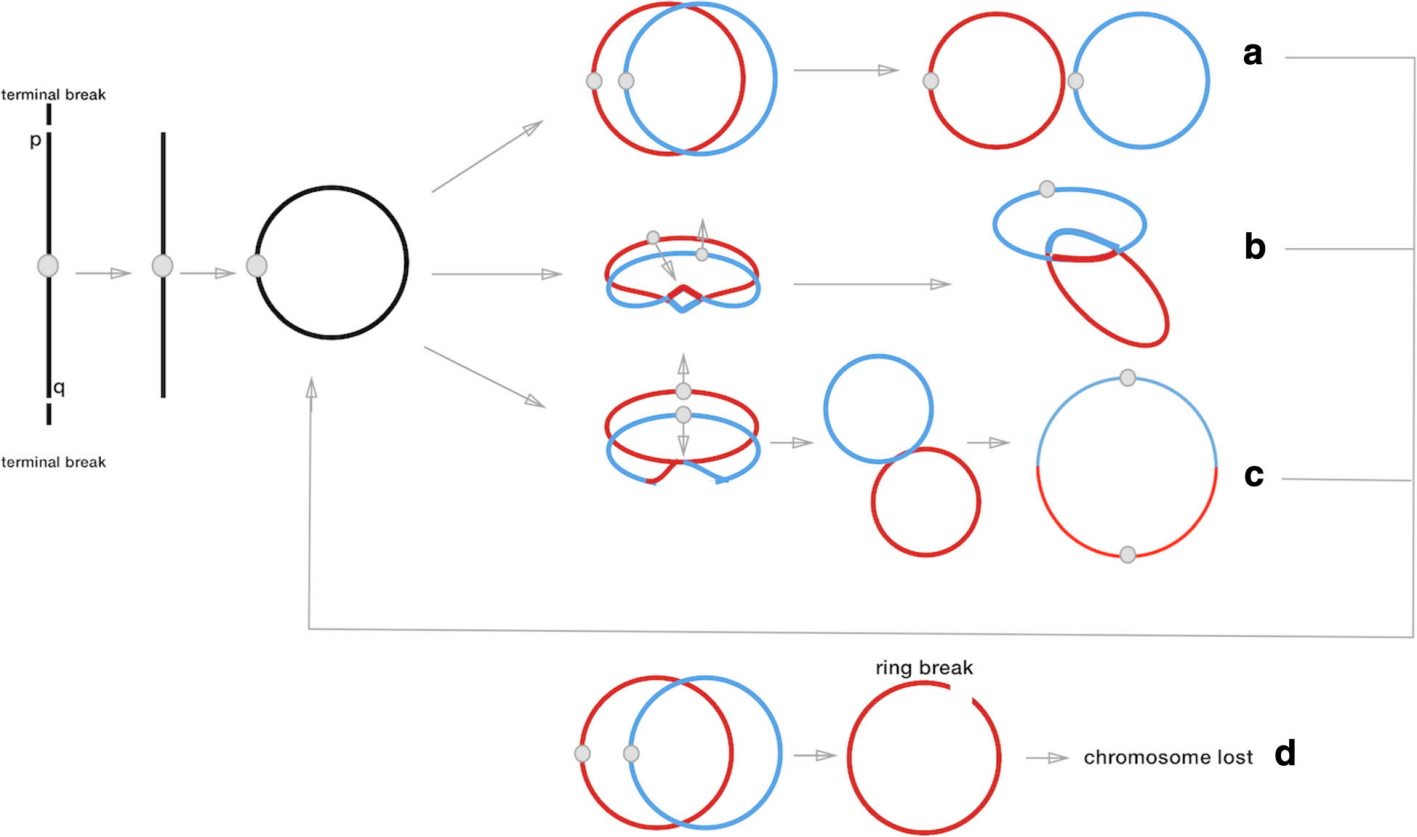
Adapted from McClintok B. 1938. Ring chromosome undergoes three pathways, **a** even or no SCE pulled in the same direction results in normal division, **b** an number of SCE pulled in the opposite results in interlocked rings, **c** and odd number of SCE caused rings to merge into a mobius-like giant ring. **d** At any point of the process’ rings may break, causing the chromosome to be degraded

The majority of ring chromosomes occur de novo where only 1% are inherited, and are formed during meiosis or after initial zygotic cell division [23, 24]. Around 90% are inherited from the mother, as ring chromosome structure impedes normal spermatogenesis, which causes infertility in males [22]. Unlike linear chromosomes, rings undergo cell division through three different pathways [25, 26]. The pathway taken is dependent on the number of sister chromatid exchanges (SCE) that occurs. No SCE or an even number of SCE’s in the same direction enables normal separation. An even number of SCEs in different directions will result in interlocked rings and an odd number of SCE leads to formation of one giant, isodicentric ring. During replication ring chromosomes are also likely to break, causing an unstable open subsequently the whole chromosome will be lost [27]. In our patient, this resulted in monosomy of chromosome 9, while, additionally, the finding of double isodicentric ring chromosome 9 (idicr(9)× 2) can easily be explained by the occurrence of non-disjunction (Fig. 7).

CGH array revealed a deletion that encompassed all *DMRT1-3* genes, in addition to *KANK1* and *DOCK8*. While *DMRT1* has been theorized to cause sex reversal, it is unclear the contribution of *KANK1* and *DOCK8* deletion in this case. A previous study has compared interstitial deletions of only KANK1 and DOCK8, but have found no overlapping phenotypic traits [28]. Sex reversal in the patient could only be explained by haploinsuficiency of *DMRT1* gene found at 9p24.3 loci. *DMRT1* is one of three types of genes that belongs to the DMRT family. From the three aforementioned genes, *DMRT1* is exclusively expressed in the genital ridge and Sertoli cells [5]. The gene plays a vital role in regulating genes for Sertoli cell differentiation, germ cell differentiation, tight junction dynamics, cell cycle control, and pluripotency in vivo [29]. Studies by Lindeman, RE et al. [30] and Matson et al. [31], showed that inactivation of *DMRT1* gene in mouse Sertoli cells induces postnatal feminization of the testis characterized by trans-differentiation of Sertoli cells into granulosa cells. The authors suggest that mouse *Dmrt1* not only plays a role in sex determination but also maintains it by repressing multiple female-promoting genes such as Foxl2, and by inducing male-promoting factors such as *SRY*. In contrast, deletions of *DMRT1* in humans, are associated with congenital feminization and even sex reversal, suggesting a similar but different control mechanism in humans [30–32]. In our case, *DMRT1-3* haploinsufficiency seemed to cause sex reversal.

## Conclusion

The presented case of 46,XY female had karyotype of mos 45,XY,-9[8]/46,XY,r(9)[29]/47,XY,+idic r(9)× 2[1]/46,XY,idic r(9)[1]/46,XY[1] and lost one copy of *DMRT1* gene. Her clinical presentation mimicking Turner syndrome should therefore highlight the importance of cytogenetic studies to detect the possibility of ring chromosome 9 in such cases. FISH is a practical yet powerful cytogenetic examination that determines the number of targetted genes, especially in mosaic cases. *DMRT1* is an important gene that plays a vital role in both sex determination and sex differentiation. This study adds a new case of haploinsufficiency of *DMRT1* causing complete XY sex reversal, which is rarely found in 9pter deletions.

## Abbreviations

BMI: Body Mass Index
CGH: Comparative Genomic Hybridization
*DMRT1*: Doublesex and Mab-3 Related Transcription factor 1
DSD: Disorders of Sexual Development
FISH: Fluorescent In Situ Hybridization
ISCN: International System for Human Cytogenetic Nomenclature
MLPA: Multiplex Ligation-dependent Probe Amplification
SCE: Sister Chromatid Exchange
SID: Sensory Integration Disorder
TS: Turner Syndrome
